# Spurious Wines

**Published:** 1887-08

**Authors:** 


### SPURIOUS WINES.

It is astonishing what an amount of acids, gums and dye stuffs, are converted into palatable wines, and relished by the unsuspecting drinkers. Fortunes are being made in the business, and the health of the consumers ruined by the gigantic frauds in this line of trade. The opening wedge of the investigation of fraudulent medicines, is being driven in by hard knocks from the aggrieved parties, and some such aggressive policy is necessary, to stem the tide of adulteration and fraudulent manufacture of wines. Talks with various persons conversant with the subject have disclosed a lamentable lack of honesty in the preparation of medicinal wines and beverages. It is more the rule than the exception, for port wine to be composed of cider, syrup, gum kino and tartaric acid, and for claret to be made from a decoction of orris root, water, raspberry juice syrup and cochineal, while most of the sherry wine on the market is a combination of cheap materials colored with alkanet root. To bring up "flat" wine a common practice is to drop a few rats into a cask through the bung hole. The rat flavor is said to be "perfectly delicious," but the sellers are careful not to sample it, leaving that delightful (?) privilege for the innocent buyers. Much of the imported stuff is hardly suitable for the swill tub, much less to be sold over the counter for patients and table use. Artificial wines are manufactured extensively, and sold either alone or in admixtures with a certain proportion of genuine wine.

Five thousand gallons of the soakings of dried peaches and raisins of doubtful record, and other superannuated vegetable substances, have just been diverted by the health authorities of this city, from a number of human stomachs and doomed, it is to be hoped, to the sewers. The intent was to color and flavor the stuff variously, to represent different kinds of wine and palm it off on the community as such. But although these 5,000 gallons may miss their mark, they form but a small installment of the concoction thus manufactured, which reaches, for the most part, its intended destination. Salicylic acid, is declared upon competent medical authority, to be injurious to health, and its use is prohibited in France.

The wholesale adulteration of California wine has long been carried on here, under the very eyes of the State Vinicultural Commission. Conclusive evidence of this fact was furnished recently, in the arrival of 150 casks of cherry juice from Hamburg. Extraordinary efforts were made to conceal the names of the consignees, but they were found to be three large commission brokers, who undoubtedly have been selected to cover the tracks of the makers of spurious wines. Several large winemakers ship directly to New York as exporters of cherry juice. Samples of the juice heretofore imported, have been analyzed and found to contain large quantities of aniline dye that is poisonous to the human system. The wine-makers here use the juice to give color and body to the thin California wine, that is known as "strawberry wine." The original Coombs "Pure Wine" bill laid heavy penalties for this adulteration with cherry juice, but the Vinicultural Commission cut out that clause in the bill.

We have frequently called attention to the unpardonable offense of adulterating food, but we know of no punishment too severe for the dastard, who would be guilty of palming off a poisonous imitation of wine upon the sick, and yet it is done every day by houses that lay claim to public confidence.

There is no one thing which so loudly calls for prohibitory legislation as the adulteration of food. Let it not be neglected.

